# Enhanced CD8 T Cell Responses through GITR-Mediated Costimulation Resolve Chronic Viral Infection

**DOI:** 10.1371/journal.ppat.1004675

**Published:** 2015-03-04

**Authors:** Maria Fernanda Pascutti, Sulima Geerman, Edith Slot, Klaas P. J. M. van Gisbergen, Louis Boon, Ramon Arens, Rene A. W van Lier, Monika C. Wolkers, Martijn A. Nolte

**Affiliations:** 1 Sanquin Research, Department of Hematopoiesis, Amsterdam, The Netherlands; 2 Landsteiner Laboratory, Academic Medical Centre, University of Amsterdam, Amsterdam, The Netherlands; 3 Bioceros BV, Utrecht, The Netherlands; 4 Department of Immunohematology and Blood Transfusion, Leiden University Medical Center, Leiden, The Netherlands; Nationwide Children’s Hospital, UNITED STATES

## Abstract

Chronic infections are characterized by the inability to eliminate the persisting pathogen and often associated with functional impairment of virus-specific T-cell responses. Costimulation through Glucocorticoid-induced TNFR-related protein (GITR) can increase survival and function of effector T cells. Here, we report that constitutive expression of GITR-ligand (GITRL) confers protection against chronic lymphocytic choriomeningitis virus (LCMV) infection, accelerating recovery without increasing pathology. Rapid viral clearance in GITRL transgenic mice coincided with increased numbers of poly-functional, virus-specific effector CD8+ T cells that expressed more T-bet and reduced levels of the rheostat marker PD-1. GITR triggering also boosted the helper function of virus-specific CD4 T cells already early in the infection, as was evidenced by increased IL-2 and IFNγ production, and more expression of CD40L and T-bet. Importantly, CD4-depletion experiments revealed that the expanded pool of virus-specific effector CD8 T cells and the ensuing viral clearance in LCMV-infected GITRL tg mice was entirely dependent on CD4 T cells. We found no major differences for NK cell and regulatory T cell responses, whereas the humoral response to the virus was increased in GITRL tg mice, but only in the late phase of the infection when the virus was almost eradicated. Based on these findings, we conclude that enhanced GITR-triggering mediates its protective, anti-viral effect on the CD8 T cell compartment by boosting CD4 T cell help. As such, increasing costimulation through GITR may be an attractive strategy to increase anti-viral CTL responses without exacerbating pathology, in particular to persistent viruses such as HIV and HCV.

## Author Summary

The ability of the immune system to rapidly respond to a viral infection is a prerequisite for the survival of an individual. The immediate reaction of innate immune cells and the subsequent response of antigen-specific lymphocytes is usually effective for rapid neutralization and removal of the invading virus. Yet, such protective immune responses need to be well controlled, as they can cause severe tissue damage that may disable the host more than the infection itself. One way that has evolutionarily been proven effective to deal with this balancing act between protective immunity and prevention of immunopathology is to render virus-specific T cells “exhausted” when the virus cannot be eradicated and the host becomes chronically infected. Exhausted T cells progressively lose their ability to kill other cells and produce different cytokines. The benefit of this exhausted state of anti-viral immunity is that it induces less tissue damage, but the downside is obviously less efficient control over the viral infection. Many immunotherapeutic and vaccination strategies against chronic viral infections are currently dedicated to overcome the exhausted state of the virus-specific T cells and thereby clear the virus. However, the accompanying risk is an exaggerated immune response with overt immunopathology. Here we describe in a mouse model that enhanced triggering through the costimulatory molecule GITR on T cells is able to provide protection upon viral infection and clear an otherwise persistent virus, but importantly without the development of collateral damage due to immunopathology. We show that GITR-mediated costimulation enhances a protective CD8 T cell response, for which CD4 T cell help is required. Our study provides new insights in how a particular costimulatory pathway can be utilized to boost anti-viral immunity, which is highly relevant for the development of safe immunotherapeutic strategies against chronic viral infections in humans.

## Introduction

The adaptive immune system has evolved to detect and remove virally infected cells. However, multiple viruses, such as human immunodeficiency virus (HIV), hepatitis C virus (HCV) or hepatitis B virus (HBV) have acquired successful counter-measures to escape from anti-viral immunity, thereby preventing complete clearance and leading to chronic and harmful infections. Cellular immunity against these viruses has been thoroughly investigated, but safe ways to boost immunity to achieve full viral elimination have yet to be developed. Apart from the emergence of viral escape mutants, three important challenges must be tackled to allow for the successful engineering of such anti-viral treatments. Firstly, prolonged exposure to viral antigens leads to functional “exhaustion” of antigen-specific T cells, which is characterized by a progressive loss of effector functions, such as cytotoxicity and the ability to simultaneously produce multiple cytokines (reviewed in [1]). This strongly contributes to decreased protection against the pathogen and is difficult to overcome by subsequent (immuno)therapy. Secondly, boosting adaptive immunity may lead to a detrimental inflammatory response and could cause life-threatening immunopathology [2]. Thirdly, this stimulation can also break the delicate immunological threshold for self-tolerance and may thereby lead to autoimmunity [3]. Thus, successful stimulation of protective immune responses against chronic viral infections requires that exhausted T cell responses are boosted without exacerbating pathology or inducing autoimmunity.

The LCMV model has proven to be a relevant model to study persistent viral infections. T cell dynamics and T cell exhaustion were initially characterized in this system [4] and later extended to a variety of human persistent infections, including HIV [5]. Chronic LCMV infection is characterized by the inability of host immune components to rapidly control the virus and the development of exhausted T cells [6,7]. Nevertheless, CD8 T cell responses and antibody responses are critical in this chronic infection model to eventually reduce LCMV titers below detection levels, and both antiviral responses are dependent on help from CD4^+^ T cells [8,9]. Interestingly, although T cell exhaustion results in impaired viral clearance, it may also be essential to prevent overwhelming damage to host tissues [10–12]. Early after infection, the ensuing T cell response to LCMV infection mediates destruction of splenic architecture that is characterized by depletion of macrophages from the marginal zone and follicular dendritic cells. This in turn leads to loss of integrity of B cell follicles, thereby delaying the induction of protective anti-viral antibody response [13,14]. Thus, control and eventual clearance of chronic LCMV is dependent on a fine balance between effective adaptive responses and prevention of immunopathology.

Costimulatory molecules are promising candidates for immunotherapy, as they are key modulators of T cell responses. TNFR superfamily members, such as CD27, OX-40, 4–1BB and GITR positively regulate the survival, proliferation and function of CD4^+^ and CD8^+^ T cells during immune activation (reviewed in [15–17]). In particular, GITR may be a promising candidate for the task of fostering a “balanced” boosting of T cell responses during chronic infections, given its well documented effects on effector and regulatory T cell biology. GITR and GITRL expression are coordinately regulated during immune responses: GITR is expressed at low levels on naïve T cells, up-regulated upon activation and maintained on CD4^+^ and CD8^+^ effector T cells, regulatory T cells (Tregs), follicular T helper cells (Tfh) and regulatory follicular T helper cells (Tfr) (reviewed in [18,19]). GITR’s unique ligand, GITRL, is temporarily expressed on activated APCs, such as DCs, B cells and macrophages [20–23]. GITR ligation on T cells *in vitro* with endogenous or recombinant GITRL, mGITRL transfected cells, or agonist anti-GITR antibodies enhances IL-2Rα (CD25), IL-2 and IFNγ expression, cell proliferation and survival, especially in the context of a sub-optimal TCR signal [22,24–27]. A protective role for GITR-mediated costimulation in T cell immunity was shown in experimental cancer therapy settings, in which GITR triggering enhanced CD8 T cell responses to tumor antigens with no or only limited autoimmunity [28–30]. GITR stimulation *in vitro* also increases Treg numbers, enhances IL-10 production, and augments their suppressive capacity, which may contribute to immune homeostasis *in vivo* [31,32]. Our previous studies demonstrated that *in vivo* GITR stimulation through transgenic expression of its natural ligand on B cells increased the cell numbers of both effector and regulatory CD4^+^ T cells in steady state conditions [33]. GITR triggering regulated the functional balance between these two populations as evidenced by a functional gain in cytokine production in the effector population, with a simultaneous expanded Treg population that retained their suppressive capacity. We tested the functional consequence of increased numbers of both regulatory and effector T cells in the experimental autoimmune encephalomyelitis (EAE) model and found a significant delay of disease onset in GITRL transgenic (tg) mice [33]. These findings imply that enhanced triggering of GITR through its natural ligand *in vivo* is protective rather than harmful, as it regulates the functional balance between regulatory and effector T cells. This concept was corroborated in a different mouse model where GITRL was overexpressed on MHCII-expressing cells [34].

Given the ability of GITR to stimulate adaptive immunity without enhancing immunopathology, we examined the impact of increased costimulation through GITR during chronic viral infection with LCMV. We found that B cell-specific GITRL tg mice infected with LCMV Clone 13 recovered from pathology and eliminated the virus faster than their WT counterparts, in a CD4^+^ T cell-dependent manner. Boosting GITR-signaling resulted in a more “acute-like” infection, with a quantitative and qualitative increase in virus-specific T cells. These studies provide insights into the regulation of a chronic viral infection by the GITR/GITRL axis and it provides a rationale for therapeutic interventions aimed at improving clearance of chronic viral infections.

## Results

### GITRL-transgenic mice are protected from chronic infection with LCMV

**Fig 1:**
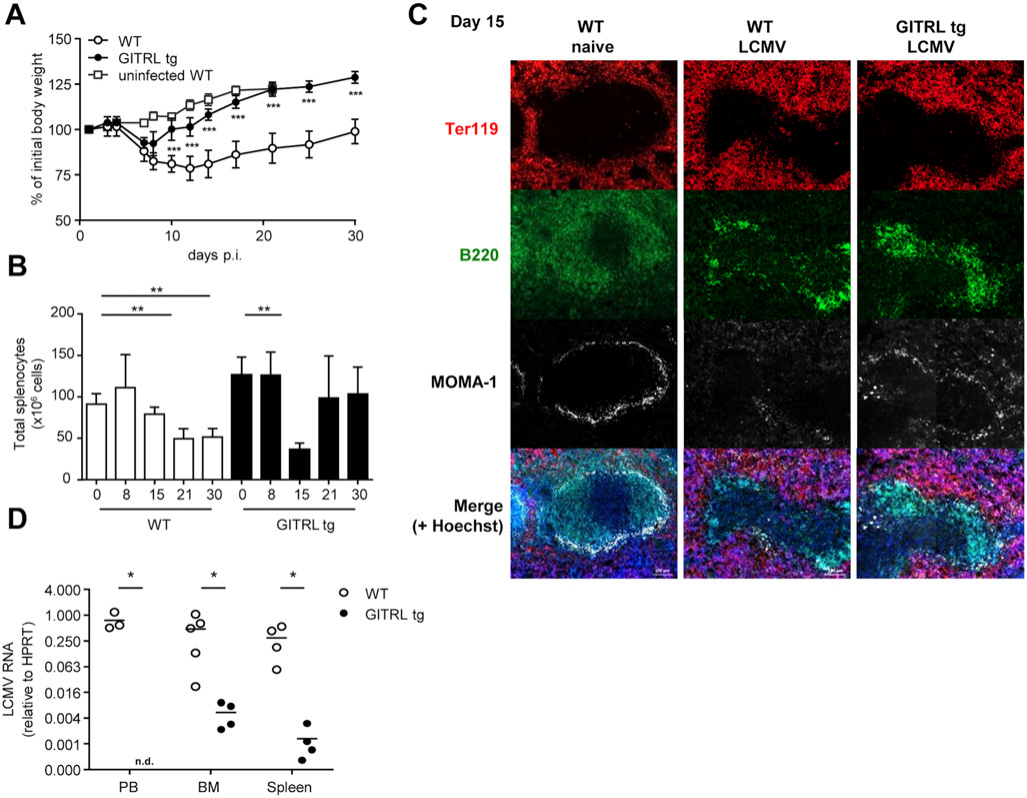
GITRL tg mice are protected against chronic LCMV infection. WT (in white) and GITRL tg (in black) mice were intravenously infected with 2×10^6^ PFU of LCMV Cl13. (A) Loss of body weight was measured throughout infection as a marker for disease severity and plotted relative to the starting weight at day 0. Uninfected WT mice are indicated by white squares. (B) Absolute numbers of nucleated spleen cells was measured throughout the infection. (C) Spleens from WT and GITRL tg mice 15 days p.i were stained for B cells (B220, green), erythrocytes in the red pulp (Ter119, red) and marginal metallophilic macrophages (MOMA-1, white), together with a nuclear staining (Hoechst, blue). The scale bar within each image corresponds to 100 μm. Each image is an automatic composite of 4 stiched pictures. (D) Viral loads were measured by RT-qPCR in peripheral blood (PB), bone marrow (BM) and spleen at day 30 post-infection (p.i.). Results are shown as LCMV RNA levels relative to HPRT RNA levels for each individual mouse. Data are representative of 2 experiments with 4–5 mice per group. Error bars represent standard deviation. *p < 0.05, **p < 0.01, ***p < 0.001.

To investigate the impact of enhanced costimulation through GITR on a chronic viral infection, we infected WT and GITRL tg mice with LCMV Clone 13. LCMV Cl13 infection induces severe immunopathology that is characterized by extensive weight loss within the first two weeks post infection (p.i.), primarily due to the anti-viral immune response [35]. While infection-induced weight loss was comparable for both mouse strains during the first week, GITRL tg mice rapidly regained their body weight during the second week of the infection, whereas WT littermates did not recover and remained below their initial weight until the end of the experiment at day 30 p.i. (Fig. 1A). This was also reflected by the gradual decline of spleen cellularity in WT mice during the course of the infection, while GITRL tg mice quickly recovered from a significant drop in splenocyte numbers by day 15 p.i. (Fig. 1B). Examination of splenic architecture at this time-point showed, as expected, that LCMV infection in WT mice induced depletion of MOMA-1^+^ marginal metallophilic macrophages and disintegration of the B cell follicles in the white pulp (Fig. 1C). Interestingly, the integrity of the marginal zone and architecture of the white pulp was also affected in GITRL tg mice, but less severe than in WT mice (Fig. 1C). Finally, at day 30 p.i. GITRL tg mice had undetectable viral loads in peripheral blood, and strongly reduced viral loads in bone marrow and spleen compared to WT mice (88-fold, p<0.01, and 233-fold, p<0.05, respectively; Fig. 1D). In summary, GITRL tg mice showed accelerated recovery from chronic LCMV infection and a strongly increased viral clearance without increased pathology.

### GITRL tg mice do not require sustained Tfh differentiation or germinal center B cell responses for increased Ab responses

**Fig 2:**
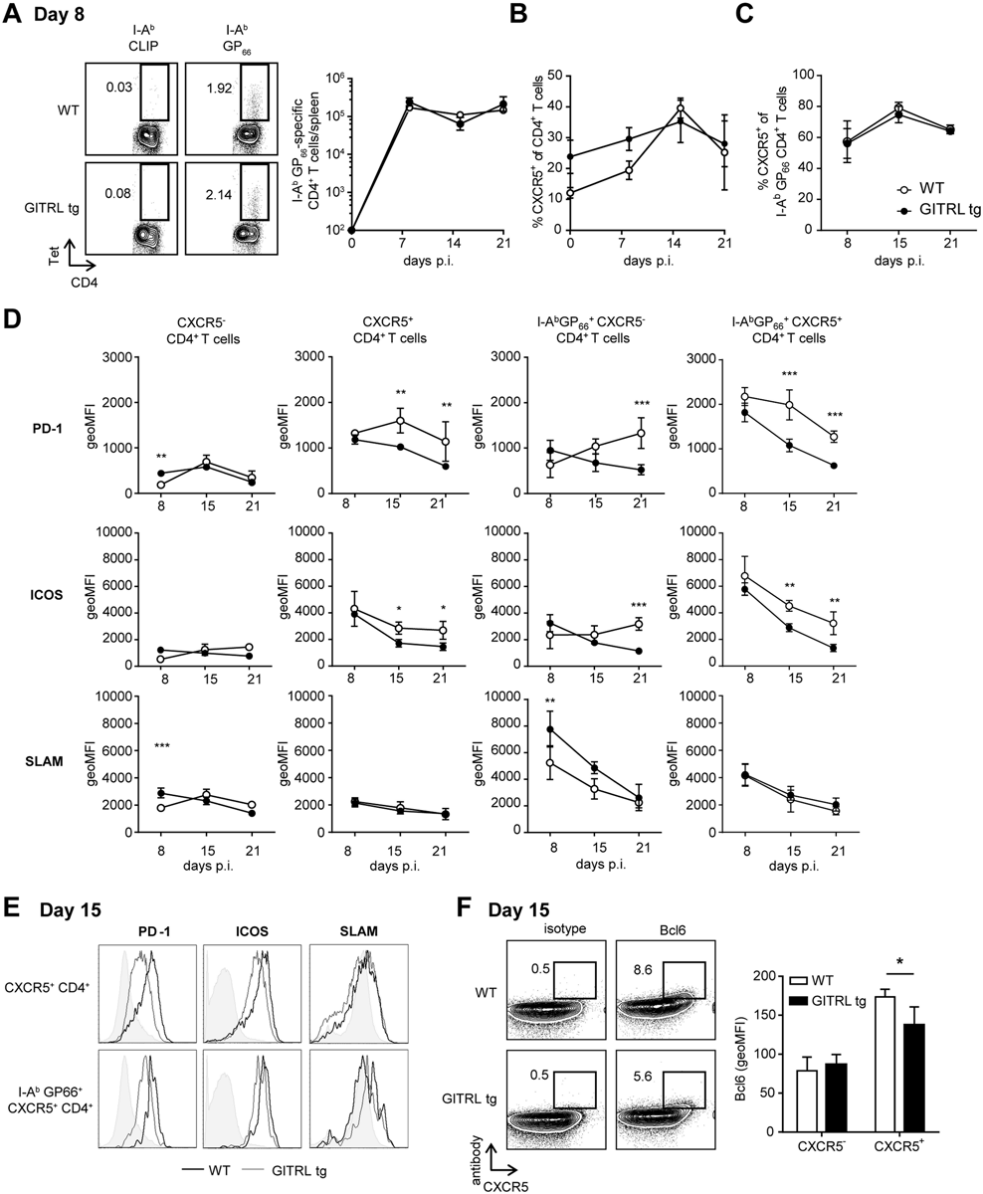
Kinetic analysis of Tfh cell responses against LCMV in GITRL tg mice. WT (in white) and GITRL tg (in black) mice were intravenously infected with 2×10^6^ PFU of LCMV Cl13. (A) *Left:* Representative FACS plots of I-A^b^CLIP-loaded control tetramers and I-A^b^GP_66_ LCMV tetramer at day 8 p.i (cells are pre-gated on CD4^+^CD3^+^ T cells). Numbers represent % of CD4^+^ T cells in the indicated gate. *Right:* Absolute number of GP_66_-specific CD4 T cells per spleen. (B) Percentage of CXCR5^+^ cells from all CD4^+^CD3^+^ T cells in spleen on the indicated days p.i. (C) Percentage of CXCR5^+^ cells from the fraction of I-A^b^GP_66_ tetramer^+^ CD4^+^CD3^+^ T cells. (D) Geometric Mean Fluorescent Intensity (MFI) of PD-1, ICOS and SLAM/CD150 on (*from left to right*): all CXCR5^-^CD4^+^CD3^+^ T cells, all CXCR5^+^CD4^+^CD3^+^ T cells, I-AbGP66 LCMV tetramer+ CXCR5-CD4+CD3+ T cells I-AbGP66 LCMV tetramer+ CXCR5+CD4+CD3+ T cells in spleens of mice during the course of the infection. (E) Representative FACS plots from (D) showing expression of PD-1, ICOS and SLAM/CD150 on total Tfh cells (top) and LCMV-specific Tfh cells (bottom) in WT (thick line) and GITRL tg mice (thin line) in spleens at day 15 p.i. (F) *Left:* Representative FACS plots showing expression of CXCR5 and an isotype control (right) or Bcl6 (left) on CD3^+^CD4^+^ T cells fom WT (top) and GITRL tg mice (bottom) in spleens at day 15 p.i. *Right:* Geometric MFI of Bcl-6 I CXCR5- and CXCR5^+^ CD4^+^ CD3^+^ cells. Data are representative of 3–5 mice per group per time point. Error bars represent standard deviation. *p < 0.05, **p < 0.01, ***p < 0.001.

Because GITR triggering increases cell numbers and function of effector CD4^+^ T cells [33,34], we assessed whether the increased protection of GITRL tg mice against chronic LCMV correlated with enhanced CD4^+^ T cell responses. We first examined the dynamics and phenotype of ensuing CD4^+^ T cell response in GITRL tg mice. We found similar numbers of GP66-specific CD4^+^ T cells in WT and GITRL tg mice at different time points during the first three weeks of infection (Fig. 2A), indicating that the overall induction of anti-viral CD4^+^ T cell responses was unaltered.

A recent study suggested that CD4^+^ T cells progressively differentiate towards Tfh cells during chronic LCMV infection to sustain antibody responses and control the virus [36]. To determine whether the constitutive GITRL expression altered the levels of Tfh cells, we examined the expression of CXCR5 on the CD4^+^ T cell population in LCMV infected mice. Even though the levels of CXCR5^+^ CD4^+^ T cells were increased before and at day 8 p.i. in GITRL tg mice, this difference was absent from day 15 p.i. onwards (Fig. 2B). Of note, the proportion of CXCR5^+^ GP66-specific CD4^+^ T cells did not differ between GITRL tg and WT mice (Fig. 2C). Besides CXCR5, expression of several surface molecules has been used to identify Tfh cells, including high expression levels of ICOS, PD-1 and Bcl6 and low expression of SLAM [37]. However, the expression level of these molecules can also reflect recent activation and have been shown to be modulated during chronic LCMV infection [36,38]. Analysis of the phenotype of total and GP66-specific CXCR5^+^ CD4^+^ T cells revealed that, irrespective of antigen specificity, CXCR5^+^ CD4^+^ T cells expressed higher levels of PD-1 and ICOS and lower levels of SLAM than CXCR5^-^ CD4^+^ T cells (Fig. 2D and E) and these levels were even higher in LCMV-specific CXCR5^+^ CD4^+^ T cells. Interestingly, while the expression of PD-1 and ICOS decreased over time, the total and GP66-specific CXCR5^+^ CD4^+^ T cells of WT mice retained higher levels of these molecules than those of GITRL tg mice, and expressed higher levels of Bcl6, suggesting either a reduced Tfh differentiation and/or reduced activation in the transgenic mice (Fig. 2D, E and F). Moreover, the kinetics of Tfh marker expression correlated with that of the B cell response. In WT mice, the numbers of germinal center B cells increased during the course of the infection, while GITRL tg mice gradually lost this cell population (S1A–S1B Fig.). In concert with this finding, GITRL tg mice also had a strongly reduced fraction of B220^lo^ CD138^+^ plasma cells compared to their WT littermates at day 30 p.i. (S1C Fig.). Yet, analysis of LCMV-specific IgG revealed that the humoral immune response to the virus was enhanced in GITRL tg mice compared to WT mice, albeit late during infection (S1D Fig.). Together, these data support previous observations of sustained Tfh and germinal center B cell response in WT mice, and further show that enhanced GITR costimulation overrides the escalation of Tfh responses, while it enhances the generation of virus-specific antibody responses at late time points after infection.

### The magnitude of regulatory T cell responses is not altered in GITRL tg mice during chronic LCMV infection

Given that Treg cells express high levels of GITR [39] and that this population is expanded and fully functional in GITRL tg mice in uninfected mice [33], we followed the proportion of FoxP3^+^ cells within the CD4^+^ T cell compartment in LCMV-infected GITRL tg and WT mice. Similar to what we found for CXCR5^+^ T cell responses, percentages of FoxP3^+^ cells were higher prior to infection, and at day 8 pi in GITRL tg mice compared to their WT counterparts. However, T reg numbers were equally high in WT and GITR tg mice by day 15 pi (S2A–S2B Fig.). We also examined the levels of CXCR5 expression in FoxP3^+^ cells, as a measurement of their ability to migrate into B cell follicles and interact with B cells. In both WT and GITRL mice, FoxP3^+^ CD4^+^ T cells expressed lower levels of CXCR5 than FoxP3^-^ CD4^+^ T cells. We found no difference in the percentages of FoxP3^+^ CXCR5^+^ CD4^+^ T cells between the two groups of mice (S2C Fig.). Thus, the initially increased numbers of Treg cells in GITRL tg mice did not lead to an enhanced expansion of this population during chronic LCMV infection and we found no indication of increased interaction between Treg and B cells in the GITRL tg mice. Finally, as GITR-triggering can modulate CD25 expression on T cells [22], we followed the expression of CD25 on FoxP3^+^ CD4^+^ T cells, which peaked at day 8 pi and subsequently declined (S2D Fig.). In line with our previous work [33], Tregs from GITRL tg mice have lower levels of CD25 expression than WT mice, though there was no difference in the kinetics (S2D Fig.). These data indicate no major differences in the magnitude of regulatory T cell responses between WT and GITRL tg mice during chronic LCMV infection.

### Effector CD4^+^ T cell function is enhanced in GITRL tg mice

**Fig 3:**
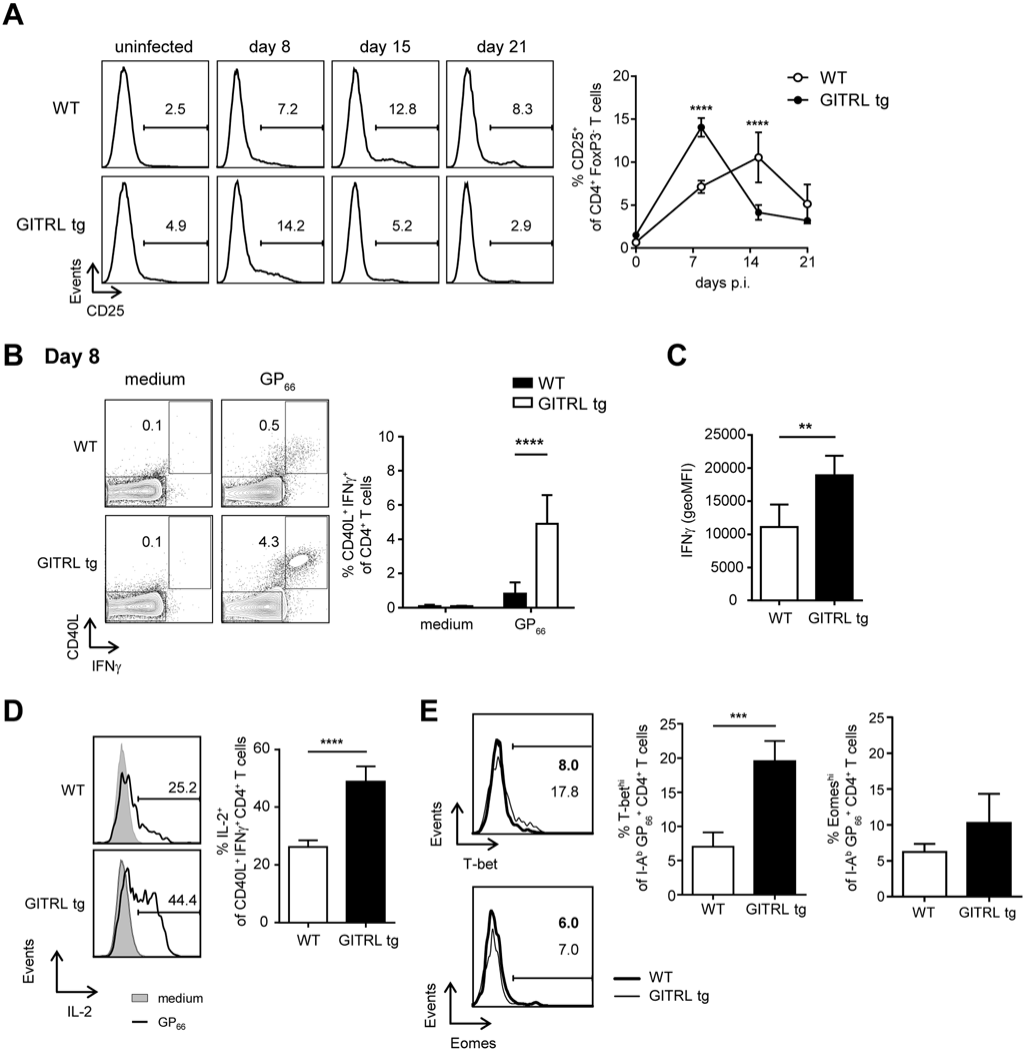
CD4^+^ T cells from GITRL tg mice are activated earlier and are more polyfunctional during chronic LCMV infection. WT (in white) and GITRL tg (in black) mice were intravenously infected with 2×10^6^ PFU of LCMV Cl13. (A) *Left*: Representative histograms showing expression of CD25 on CD3+ CD4+ FoxP3- cells before and at different times p.i for WT (top) and GITRL tg (bottom) mice. *Right*: Percentage of CD25^+^ cells among CD3^+^ CD4^+^ FoxP3^-^ cells. (B) *Left*: Representative FACS plots showing expression of IFNγ and CD40L in CD3^+^ CD4^+^ cells from WT (top) and GITRL tg (bottom) mice, after culture of total splenocytes in medium alone (left) or with the CD4-restricted peptide GP_66_ (right) for 4 hours in the presence of Brefeldin A. Splenocytes were obtained 8 days p.i. *Right*: Percentage of CD40L^+^ IFNγ^+^ cells within the CD3^+^ CD4^+^ cells. (C) geometric MFI of IFNγ on CD40L^+^ IFNγ^+^ CD3^+^ CD4^+^ cells. (D) *Left*: Representative histograms showing expression of IL-2 on CD40L^+^ IFNγ^+^ CD4^+^ cells for cells cultured in medium (black line) or with GP_66_ peptide (grey filled curve) from WT (top) and GITRL tg (bottom) mice. *Right*: Percentage of IL-2^+^ cells within the CD40L^+^ IFNγ^+^ CD3^+^ CD4^+^ cells. (E) *Left*: Representative histograms showing expression of T-bet (top) and Eomes (bottom) on I-A^b^GP_66_ tetramer^+^ CD4^+^CD3^+^ T cells from WT (black line) or GITRL tg mice (grey filled curve) at day 8 p.i. *Right*: Percentage of T-bet^hi^ (top) or Eomes^hi^ (bottom) cells within I-A^b^GP_66_ tetramer^+^ CD4^+^CD3^+^ T cells. Data are representative of 4–5 mice per group per time point. Error bars represent standard deviation. *p < 0.05, **p < 0.01, ***p < 0.001, ****p < 0.0001.

When we analyzed expression of CD25 in FoxP3^-^ CD4^+^ T cells from GITRL tg and WT mice, we found that GITRL tg mice had significantly higher levels of CD25^+^ FoxP3^-^ effector CD4^+^ T cells early after infection (day 8 p.i.), which then declined, while in WT mice the abundance of these cells peaked a week later (day 15 p.i., Fig. 3A). These results suggested that LCMV-related activation of CD4^+^ T cells was faster in the transgenic mice. We then analyzed virus-specific production of cytokines and expression of CD40L by CD4^+^ T cells at day 8 p.i. in the 2 groups of mice. Interestingly, we found a higher percentage of CD40L^+^ IFNγ^+^ CD4^+^ T cells in response to stimulation with a CD4-restricted viral peptide in GITRL tg mice (Fig. 3B). These virus-specific cells not only were increased in percentage, but they also expressed higher levels of IFNγ on a per cell basis (Fig. 3C) and contained a significantly higher proportion of cells simultaneously expressing IL-2 (Fig. 3D).

Transcription factors T-bet and Eomes have been related to CD4^+^ T cell function/exhaustion during LCMV infection, as T-bet is expressed in exhausted CD4^+^ T cells, though it is higher in functional memory T cells, whereas Eomes is rather increased in (a subset of) exhausted CD4^+^ T cells [38]. We found expression of T-bet and Eomes restricted to a subset of virus-specific CD4^+^ T cells (Figs. 3E and S3A). Total CD4^+^ T cells from GITRL tg mice contained a higher percentage of both T-bet^+^ and Eomes^+^ cells than those from their WT counterparts, which were clearly separate populations (S3B Fig.). Virus-specific CD4^+^ T cells also contained separate populations expressing only one of the two transcription factors (S3C Fig.). Interestingly, we found a strong increase in T-bet^+^ virus-specific T cells in GITRL tg mice, while Eomes^+^ virus-specific T cells were not significantly different (Fig. 3E).

In summary, virus-specific CD4^+^ T cells from GITRL tg mice are more polyfunctional than those from WT mice, which correlates with an increased expression of the transcription factor T-bet, indicating that GITR-mediated costimulation boosts the rapid induction of functional Th1 cells upon LCMV Cl13 infection.

### Virus-specific CD8^+^ T cell responses are enhanced very early during infection and correlate with accelerated viral clearance

**Fig 4:**
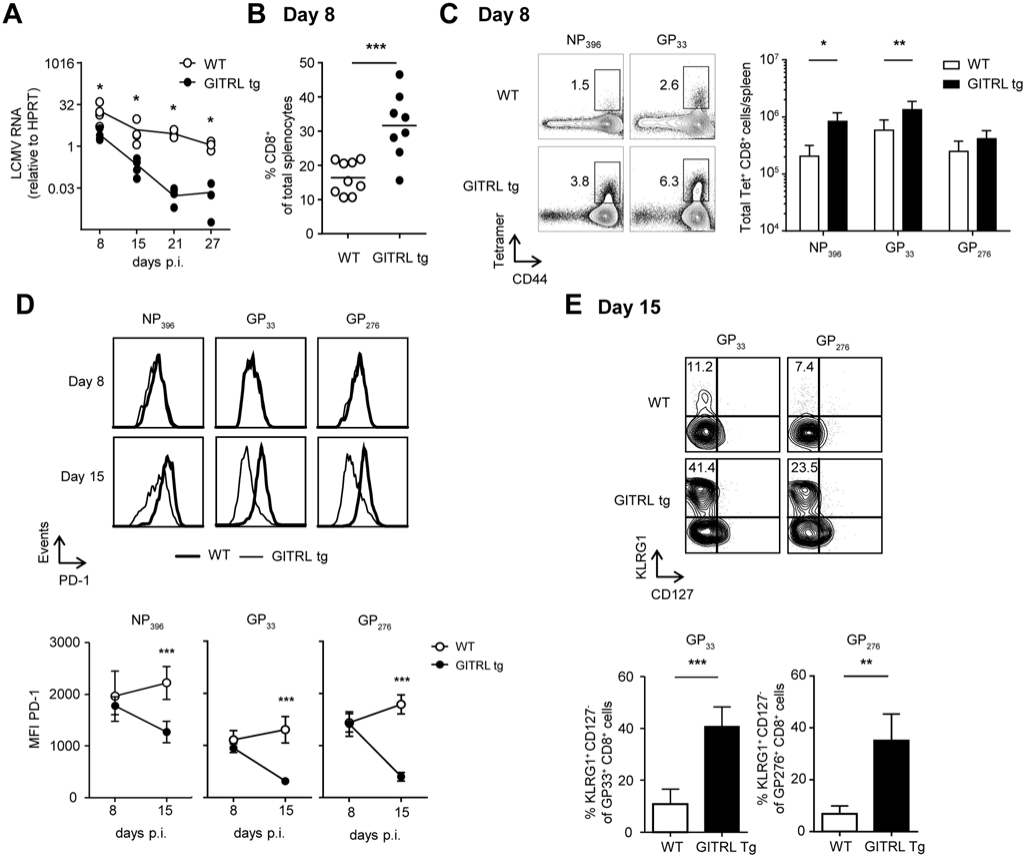
Protective anti-viral CD8 response in GITRL tg mice is established very early during infection. Analysis of CD8^+^ T cell responses in spleens from WT (in white) and GITRL tg (in black) mice at different time points after infection with 2×10^6^ PFU of LCMV Cl13. (A) Kinetic analysis of viral RNA measured by RT-qPCR. Results are shown as LCMV RNA levels relative to HPRT RNA levels for each individual mouse. (B) % of CD8 T cells from total nucleated cells in the spleen at day 8 p.i. (C) *Left*: Representative FACS stainings of MHC-I LCMV-tetramer vs CD44 on CD8^+^ CD3^+^ T cells at day 8 p.i. *Right*: Quantification of these data as absolute numbers of CD8^+^ T cells specific for the indicated LCMV-peptide per spleen at day 8 p.i. (D) *Left*: PD-1 expression on CD8^+^ CD3^+^ T cells staining positive for either the H2-D^b^NP_396_, H2-D^b^GP_33_ or H2-D^b^GP_276_ tetramer on day 8 (top row) or day 15 (bottom row) in either WT (thick line) or GITRL tg (thin line) mice. *Right*: Expression analysis of PD-1 (in MFI) on LCMV-specific CD8 T cells at days 8 and 15 in WT (open circles) and GITRL tg (black circles) mice. Data represent mean ±standard deviation of 4–5 mice per group. (E) *Left*: Representative FACS plot showing CD127 vs KLRG1 expression of H2-D^b^GP_33_ tetramer^+^ and H2-D^b^GP_276_ tetramer^+^ on CD8^+^CD3^+^ T cells at day 15 p.i. % of KLRG1^+^CD127^-^ effector CD8^+^CD3^+^ T cells is indicated upper left. *Right*: Quantification of KLRG1^+^CD127^-^ effector CD8^+^CD3^+^ T cells specific for the indicated LCMV-epitope at day 15 p.i. *p < 0.05, **p < 0.01, ***p < 0.001.

We reasoned that the early activation and polyfunctionality of virus-specific CD4^+^ T cells could also reflect on an enhanced CD8^+^ T cell response to the virus. Kinetic analysis of the viral load revealed the GITRL tg mice were able to rapidly control the infection, as at day 8 expression of viral RNA in spleens was already 7-fold lower compared to WT littermates and this difference further increased over the course of the infection, reaching a 176-fold reduction by day 21 p.i. (Fig. 4A; p<0.05). To determine whether the CD8^+^ T cell response against LCMV correlated with viral clearance in GITRL tg mice, we examined the kinetics of the anti-viral CTL response in GITRL tg mice. An overall CD8^+^ T cell expansion in GITRL tg mice was already evident at day 8 p.i. (Fig. 4B), and included a strong increase CD8^+^ T cells directed against the early and intermediate epitopes NP396 and GP33 (Fig. 4C). On day 8 p.i., expression of the rheostat marker PD-1 on the LCMV-specific CTLs was comparable. Strikingly, on day 15 p.i. the expression of PD-1 was reduced in GITRL tg mice, while it further increased in WT mice (Fig. 4D). At this time point, a larger part of the LCMV-specific CD8^+^ T cells in GITRL tg mice were KLRG1^+^CD127^-^ CD8^+^ T cells compared to those from WT mice (Fig. 4E). Cells with this phenotype were originally identified as short-lived effector CD8^+^ T cells [40], but it was recently shown that these cytotoxic cells can also be maintained after acute LCMV infection and that they are highly protective upon re-infection [41].

**Fig 5:**
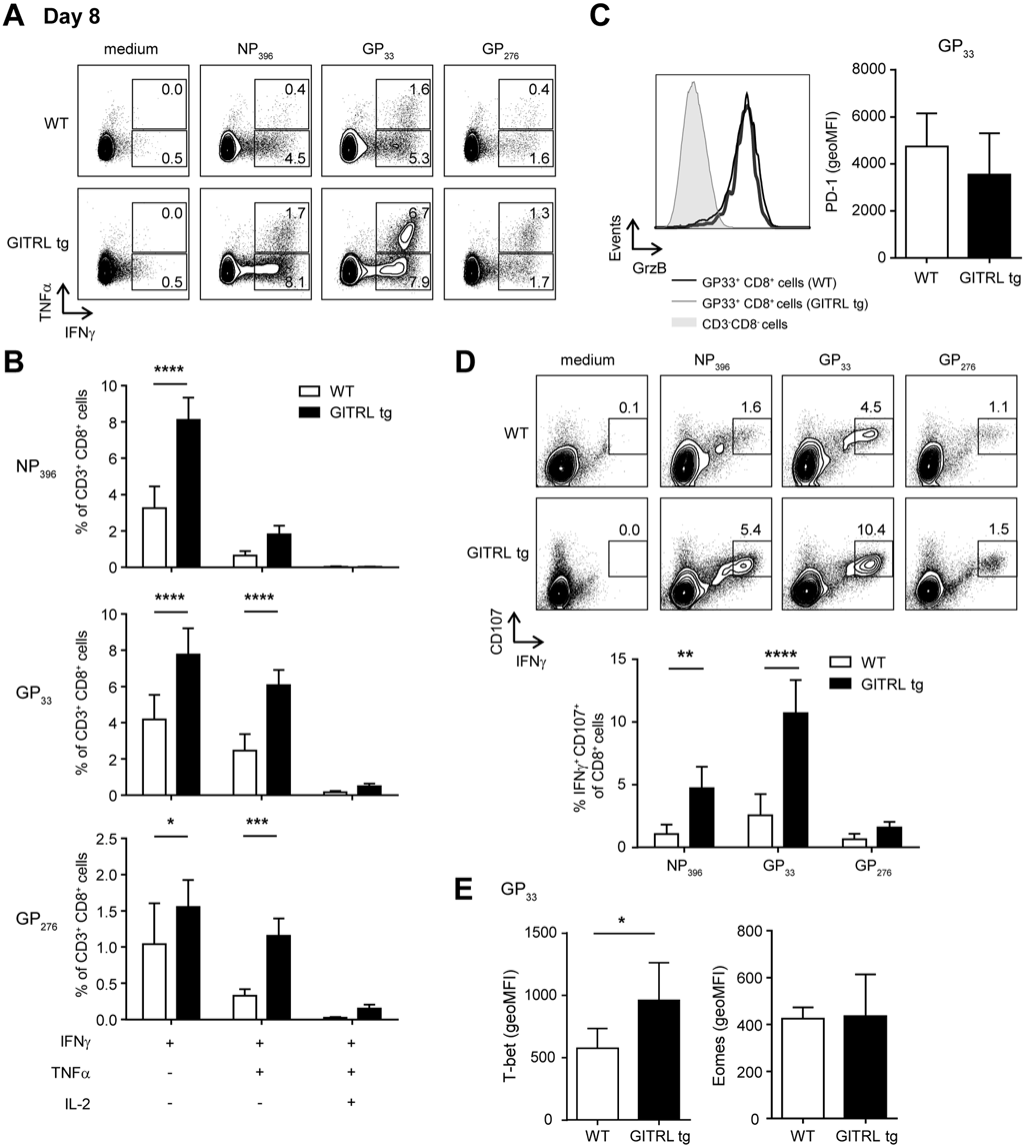
Virus-specific CD8^+^ T cells are more polyfunctional in GITRL tg mice. Analysis of functionality of virus-specific CD8^+^ T cell responses in spleens from WT (in white) and GITRL tg (in black) mice at day 8 after infection with 2×10^6^ PFU of LCMV Cl13. (A) Representative FACS plot showing IFNγ and TNFα expression in CD3^+^ CD8^+^ cells from WT (top) and GITRL tg (bottom) mice, after culture of total splenocytes in medium alone or with the CD8-restricted peptides NP_396_, GP_33_ or GP_276_ for 4 hours in the presence of Brefeldin A. (B) Analysis of polyfunctional LCMV-specific CD8 T cells by identifying the fraction of IFNγ^+^ CD8^+^ T cells that also stains positive for TNFα or TNFα and IL-2, upon restimulation with GP_33_ peptide, as indicated in (A). (C) *Left*: Representative histograms showing expression of Granzyme B on H2-D^b^GP_33_ tetramer^+^ CD8^+^CD3^+^ T cells at day 8 p.i from WT (thick line) or GITRL tg (grey filled curve). Expression of GrzB on CD3^-^ CD8^-^ cells is also shown for comparison (thin line). *Right*: geometric MFI of Granzyme B on H2-D^b^GP_33_ tetramer^+^ CD8^+^CD3^+^ T cells. (D) *Top*: Representative FACS plot showing IFNγ and CD107α/β expression in CD3^+^ CD8^+^ cells from WT (top) and GITRL tg (bottom) mice, from splenocytes cultured as in (A). *Bottom*: Percentage of IFNγ^+^ CD107α/β^+^ cells within the CD3^+^ CD8^+^ cells. (E) geometric MFI of T-bet^hi^ (left) or Eomes^hi^ (right) on H2-D^b^GP_33_ tetramer^+^ CD8^+^CD3^+^ T cells. Data are representative of 4–5 mice per group per time point. Error bars represent standard deviation. *p < 0.05, **p < 0.01, ***p < 0.001, ****p < 0.0001.

After restimulation with different CD8-restricted viral peptides, CD8^+^ T cells from GITRL tg mice contained a significantly higher percentage of IFNγ^+^ TNFα^-^ and IFNγ^+^ TNFα^+^ cells (Fig. 5A and B). Ex vivo measurement of Granzyme B expression showed that, in both groups of mice, almost all GP33^+^ CD8^+^ T cells were Granzyme B^+^, irrespective of KLRG1 expression, and there was no difference in the expression levels of this molecule between groups (Fig. 5C). However, virus-specific CD8 T cells from GITRL tg mice were more able to degranulate (as measured by CD107α/β expression) after restimulation with viral peptides (Fig. 5D). CD107α/β^+^ cells were also mostly IFNγ^+^, further demonstrating that the virus-specific CD8 T cells in GITRL tg mice are more polyfunctional (Fig. 5D). Finally, GP33^+^ CD8^+^ T cells from GITRL tg mice had a higher expression of T-bet, but similar expression of Eomes, when compared to their WT counterparts (Fig. 5E).

Together, these findings illustrate that GITRL tg mice have a greatly enhanced anti-viral CD8 T cell response, both quantitatively and qualitatively, early after infection and this coincides with faster viral clearance.

### Functionality of CD8 T cell responses is preserved in GITRL tg mice in the chronic phase of LCMV infection

**Fig 6:**
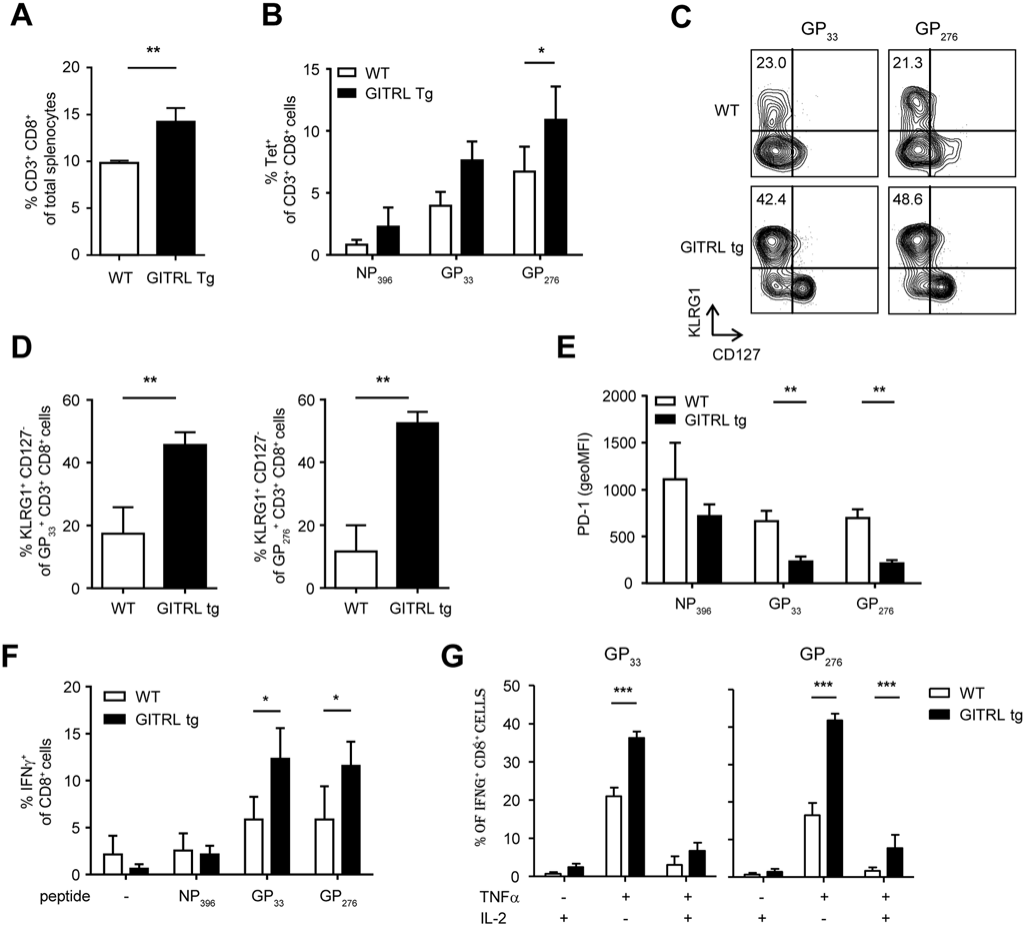
Anti-viral CD8 T cell responses are less exhausted in GITRL tg mice. Analysis of CD8 T cell responses in spleens from WT (in white) and GITRL tg (in black) mice at day 27 p.i. with 2×10^6^ PFU of LCMV Cl13. (A) % of CD8^+^CD3^+^ T cells from total nucleated cells in the spleen. (B) % of LCMV-specific CD8^+^CD3^+^ T cells from total splenocytes, identified by staining with MHC-I tetramers loaded with the immunodominant LCMV-epitopes NP396, GP33 or GP276. (C) Representative FACS plots showing KLRG1 vs CD127 expression of H2-D^b^GP_33_ tetramer^+^ (left) or H2-D^b^GP_276_ tetramer^+^ (right) CD8^+^ T cells. % of KLRG1^+^CD127^-^ effector CD8^+^CD3^+^ T cells is indicated upper left. (D) Quantification of KLRG1^+^CD127^-^ effector CD8^+^ T cells specific for the indicated LCMV-epitope, as shown in (C). (E) MFI of PD-1 expression on LCMV-specific CD8^+^ T cells staining positive with the indicated MHC-I tetramers. (F) % of CD8 T cells staining positive for IFNγ upon restimulation of total splenocytes for 6 hours with LCMV-specific peptides, in the presence of Brefeldin A for the last 4 hours. (G) Analysis of polyfunctional LCMV-specific CD8 T cells by identifying the fraction of IFNγ^+^ CD8^+^ T cells that also stains positive for TNFα and/or IL-2, upon peptide-restimulation, as indicated in (F). Data are representative of 2 experiments with 3–4 mice per group. Error bars represent standard deviation. *p < 0.05, **p < 0.01, ***p < 0.001.

High antigen levels drive CD8^+^ T cell exhaustion in chronic LCMV infection [42]. We thus examined virus-specific CD8^+^ T cell responses during the chronic phase of the LCMV infection. As seen during the acute phase, total CD8^+^ T cell numbers were significantly increased in GITRL tg mice when compared to WT mice at day 27 p.i. (Fig. 6A; p<0.01) We next measured the responses to the three immunodominant CD8-restricted epitopes, and found a trend to elevated responses in GITRL tg mice (significant for the late epitope GP276 (Fig. 6B; p<0.05). Phenotypic analysis indicated that nearly all LCMV-specific CD8^+^ T cells were CD44^hi^CD62L^-^ effector-memory T cells in both GITRL tg and WT mice (*data not shown*). Again, GITRL tg mice contained many more KLRG1^+^CD127^-^ effector CD8^+^ T cells (Fig. 6C and D). The expression levels of PD-1 on virus-specific CD8^+^ T cells were also decreased in GITRL tg mice (Fig. 6E), suggesting that these cells were more functional than their WT counterparts. Importantly, GITRL tg mice contained not only more IFNγ-producing CD8^+^ T cells, as expected from the MHC-class I tetramer stainings (Fig. 6F), but also a much higher fraction displayed a polyfunctional phenotype as determined by co-production of IFNγ with TNFα and/or IL-2 (Fig. 6G). These data thus demonstrate that virus-specific CD8^+^ T responses are protected from exhaustion in GITRL tg mice.

### Protection against viral infection in GITRL tg mice is CD4 T cell-dependent

**Fig 7:**
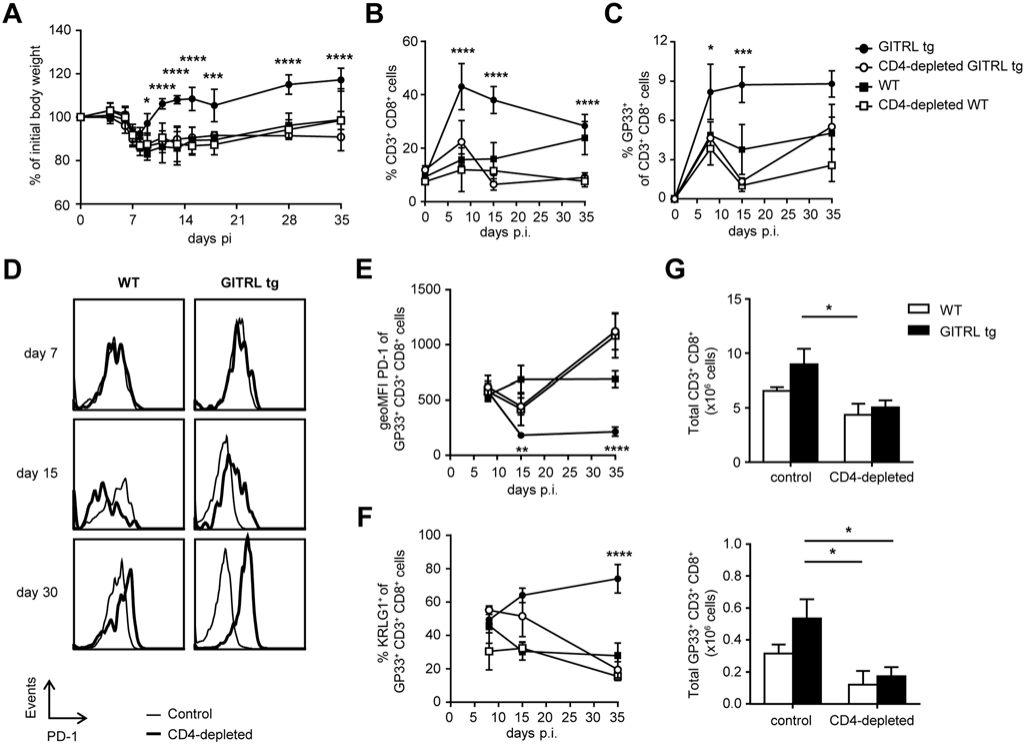
Protection and enhanced anti-viral CD8 T cell response in GITRL tg mice are CD4 T cell-dependent. Control WT (black square), GITRL tg (black circles) and WT and GITRL tg mice that were depleted of CD4 T cells by injection with anti CD4 on days-3 and 4 p.i. (CD4-depleted WT, white squares, CD4-depleted GITRL tg, white circles) were intravenously infected on day 0 with 2×10^6^ PFU of LCMV Cl13. (A) Loss of body weight was measured throughout infection as a marker for disease severity and plotted relative to the starting weight at day 0. (B) Kinetic analysis of the % of total CD3^+^ CD8^+^ T cells from all leukocytes in blood at days 0, 7, 15 and 35 p.i. To correct for the loss of CD4 T cells, data was calculated as the % of CD3^+^CD8^+^ T cells from all leukocytes minus the CD4 T cells (identified here as CD8^-^CD3^+^ cells). (C) Analysis of LCMV-specific CD8^+^ T cells staining positive for the H2-D^b^GP_33_ tetramer, represented as % of all CD8^+^CD3^+^ T cells from blood. (D) PD-1 expression on H2-D^b^GP_33_ tetramer ^+^ CD8^+^CD3^+^ cells in blood from WT (left) or GITRL tg (right) in either control (black line) or CD4-depleted (grey filled curve) mice at different days p.i. (E) Expression analysis of PD-1 (in MFI) on GP_33_-specific CD8 T cells from blood. (F) Percentage of KLRG1^+^ among GP_33_-specific CD8 T cells from blood. (G) Absolute numbers of CD8^+^ T cells (top) or GP_33_-specific CD8 T cells (bottom) per spleen at day 35 p.i. Data are representative of 4–5 mice per group per time point. Error bars represent standard deviation. *p < 0.05, **p < 0.01, ***p < 0.001, ****p < 0.0001.

Because GITR triggering increases cell numbers and function of effector CD4^+^ T cells [33,34], and because we found increased virus-specific CD4^+^ T cell function in LCMV-infected GITRL tg mice (Fig. 3), we next assessed whether the increased protection of GITRL tg mice against chronic LCMV infection required CD4^+^ T cells. GITRL tg and WT mice were injected with a depleting antibody against CD4 before and early during the infection (on days-3 and day 4 p.i.;[43]). This regimen successfully depleted the CD4^+^ T cells in the first week and prevented the recovery of body weight in GITRL tg mice in the second week of the infection with LCMV (Fig. 7A). The pattern of weight loss observed in CD4-depleted GITRL tg mice was comparable to that found in WT mice and CD4^+^ T cell depletion in WT mice did not further enhance weight loss (Fig. 7A). Analysis of viral loads revealed that depletion of CD4^+^ T cells during the initial phase of the infection greatly impaired viral clearance in GITRL tg mice. At day 30 p.i., CD4-depleted GITRL tg mice had a 1728-fold increase in viral loads, compared to non-depleted GITRL tg mice (ratio LCMV RNA over HPRT: 33.7 ± 13.2 vs 0.0195 ± 0.0334, respectively; p<0.001; n = 3 vs 5). This finding demonstrates that CD4 T cells play a critical role to control chronic LCMV infection in GITRL tg mice.

### Enhanced anti-viral CD8 T cell response in GITRL tg mice is CD4 T cell-dependent

We next examined whether the enhanced anti-viral CD8^+^ T cell response was also mediated by CD4^+^ T cells. Indeed, depletion of CD4^+^ T cells in GITRL tg mice abrogated the early increase in CD8^+^ T cell expansion during the first week of the infection and induced a crash of both the total CD8^+^ T cell pool (Fig. 7B) and the LCMV-specific CD8^+^ T cells at day 15 p.i. (Fig. 7C). While the total CD8^+^ T cell pool was maintained in CD4-depleted WT mice, virus-specific CD8^+^ T cells greatly contracted in the absence of CD4^+^ T cells (Fig. 7B and C). CD4-depletion also prevented the observed decrease of PD-1 on the remaining LCMV-specific CD8^+^ T cells from GITRL tg mice on day 15 p.i. On day 30 p.i., CD4-depleted WT and GITRL tg mice had even higher levels of PD-1 than non-depleted WT mice (Fig. 7D and E). Maintenance of KLRG1^+^ GP33^+^ CD8^+^ T cells in GITRL tg mice was also dependent on the presence of CD4^+^ T cells (Fig. 7F). Finally, these effects in peripheral blood could also be seen in the spleen, where the increase in total and virus-specific CD3^+^ CD8^+^ T cells at day 35 p.i. was also lost in the absence of CD4^+^ T cells (Fig. 7G). Together, we conclude that the protective anti-viral CD8^+^ T cell response in GITRL tg mice is fully dependent on CD4^+^ T cells, suggesting that although direct GITR triggering on CD8^+^ T cells might account for some increase in T cell function, it is not sufficient to clear the virus and prevent CD8^+^ T cell exhaustion in this model of chronic viral infection.

## Discussion

Here we describe how enhanced costimulation through GITR accelerated viral clearance during chronic LCMV infection, reduced pathology and prevented CD8^+^ T cell exhaustion. Protection from the chronic infection was CD4^+^ T cell-dependent and coincided with a strong increase in virus-specific effector CD8^+^ T cells. These data suggest that increased costimulation through GITR functionally boosted an early virus-specific CD8^+^ T cell response, leading to faster viral clearance and preventing the establishment of chronicity.

Robust and functional CD4^+^ T cell responses are critical in the generation of an effective antiviral response, as they can prevent CD8^+^ T cell exhaustion during chronic viral infections, including LCMV and HCV [9,44]. In chronic LCMV, it has been suggested that persistence of antigen drives differentiation towards a Tfh phenotype, in order to sustain antibody responses [36], which would compensate for the gradual exhaustion in CD8^+^ T cell function. Because enhanced GITR-mediated costimulation in the steady state led to increased Tfh cell numbers (Fig. 2B), we expected an increased humoral anti-viral response in GITRL tg mice. However, the number of virus-specific CXCR5^+^ CD4^+^ T cells in GITRL tg mice was similar to WT mice and their phenotype was less sustained, which coincided with a reduction in germinal center B cells and plasma cells at later time-points in GITRL tg mice. Surprisingly, this coincided with an increase rather than a decrease in virus-specific IgG levels at day 30 in GITRL tg mice compared to WT mice. As the viral loads were already contained at this time point in GITRL tg mice, this boost in anti-LCMV antibodies cannot explain the observed early protection against the virus. Instead, it is more likely that the late boost in anti-viral antibodies is a consequence rather than a cause of LCMV clearance, which fits with the concept that presence of this virus negatively affects the development of protective antibodies [45]. In conclusion, overexpression of GITRL on B cells protects against viral chronicity, but this could not be attributed to an increased humoral immune response to the virus.

In contrast to the Tfh and antibody response, we found that increased viral clearance in GITRL tg mice on day 8 coincided with more virus-specific CD8 T cells and a qualitative increase in T cell help. Although we did not find more GP66-specific CD4 T cells (Fig. 2A), GITRL tg mice did develop a rapid response of CD25^+^ FoxP3^-^ and T-bet^+^ CD4^+^ T cells early after infection and displayed a strong increase in CD4^+^ T cells expressing CD40L and producing IL-2 and IFNγ upon restimulation with viral peptide (Fig. 3). As CD4^+^ T cells play an important role in sustaining virus-specific CD8^+^ T cells during chronic LCMV infection [9], it is highly likely that the observed increase in helper function of CD4 T cell from GITRL tg mice boosts the CD8 T cell response in chronic LCMV. Indeed, GITRL tg mice developed more virus-specific CD8 T cells with an effector phenotype (KLRG-1^+^ CD127^-^), which also produced and secreted more different cytokines upon peptide restimulation than WT mice (Fig. 5). These results are in agreement with previous observations that particularly the KLRG1^hi^ effector CD8^+^ T cells were lost in chronically infected mice [46]. The increased polyfunctional cytokine response and decrease in PD-1 expression in CD8 T cells from GITRL tg mice was maintained till the end of the infection, indicating that these cells were prevented from exhaustion. Decreased PD-1 levels may be the result of increased viral clearance, as sustained PD-1 expression has been linked to persistent antigen exposure [47]. However, it may also be a direct cause of the increase in T-bet expression (Fig. 5E), as T-bet can directly repress transcription of the gene encoding PD-1 in both CD4^+^ and CD8^+^ T cells [48]. In conclusion, enhanced GITR-mediated costimulation boosts the development of effective Th1 cells upon LCMV infection and enhances and sustains a pool of highly functional virus-specific effector CD8^+^ T cells, thereby preventing the establishment of viral chronicity. Importantly, viral persistence, immunopathology and T cell function are intimately linked in the LCMV model. It is therefore likely that the observed weight gain decreased spleen pathology, late boost in antiviral antibodies and possibly also part of the T cell phenotype in GITRL tg mice is the result of lower viral loads during the infection due to the enhanced CTL function early on.

GITR-GITRL interactions can occur between different types of T cells and APCs, and it is not yet clear what the impact is of GITR-mediated costimulation on every T cell subset. We reasoned that increased GITRL expression on B cells would target CD4^+^ T cells rather than CD8 T cells, as the latter do not enter B cell follicles. Indeed, in the steady state, GITRL tg mice showed significant alterations in the CD4^+^ but not the CD8^+^ compartment [33]. Interestingly, transgenic overexpression of GITRL on MHC-II-expressing cells, i.e. macrophages, dendritic cells and B cells, also leads to very similar alterations in the CD4^+^ T cell compartment with no changes observed in the CD8^+^ T cells [34]. This would argue that, at least in the steady state, GITRL-expressing APCs mainly influences CD4^+^ T cell numbers and function. In line with this, we observed down-regulation of GITR only on CD4^+^ T cells but not in CD8^+^ T cells in the transgenic mice in the steady state (S5A Fig.). In contrast, upon LCMV infection, we found that enhanced GITRL expression on B cells strongly affected CD8^+^ T cell numbers, phenotype and function, but we found that this was fully dependent on CD4 T cells. This does not imply that GITR triggering on CD8 T cells does not play a role, but may be a reflection of the fact that CD8 T cells interact less with B cells compared to CD4 T cells. In fact, there is ample evidence from *in vitro* [26] and *in vivo* [49–51] experiments that GITR triggering has a stimulating role on the function of CD8 T cells (reviewed in [52]). Yet, the observation that protection to LCMV chronicity in GITRL tg mice was completely lost when CD4 T cells were depleted (Fig. 7), demonstrates that GITR-mediated costimulation on CD8 T cells is at least not sufficient for the protective effect. However, we cannot exclude an additive contribution of GITR triggering on the CD8 T cells in the presence of the CD4 compartment. We found no differences in GITR expression in CD4^+^ or CD8^+^ T cells during LCMV infection (S5B Fig.), which may be related to the upregulation of GITR on T cells following LCMV infection [51]. In conclusion, we postulate that GITR-mediated costimulation enhances CD8-mediated viral clearance by boosting the helper function of CD4 T cells.

Apart from the well-established role of CD8 T cells in LCMV clearance, it could be that NK cells, which express GITR, are also partly responsible for the early-enhanced viral clearance observed in the GITRL tg mice. However, several lines of evidence indicate that NK cell activation promotes pathology and chronic LCMV infection and limits CD8 T cell function, through impairment of APC function [53,54]. Analysis of NK cell numbers, maturation (DX5, CD11b and CD27 expression) and activation (KLRG1 expression) both in the steady state and during LCMV infection revealed no differences between groups (S4 Fig.), which makes it not very likely that GITR signaling in NK cells plays an important role in the observed protection against LCMV chronicity in GITRL tg mice.

Although we postulate that the protected phenotype of GITRL tg mice to LCMV infection is due to direct GITR-mediated costimulation on the virus-specific T cells, it could be that pre-existing differences between WT and GITRL tg mice prior to the infection also have an effect. As described previously, GITRL tg mice have more effector and regulatory CD4^+^ T cells in the steady state [33], though this phenotype is age-dependent and not yet very pronounced in the young mice (~5 weeks old) we use for LCMV infection. Although we did find somewhat increased numbers of Tregs in young GITRL tg mice prior to infection (S2B Fig.), it is unlikely that they play an important role in the early phase of the infection, as Tregs lose their suppressive capacity upon exposure to type I IFNs [55]. Chronic LCMV infection has been shown to expand Tregs due to the expression of endogenous retroviral superantigens, but this only occurs in the late phase of the response [56]. We observed that GITRL tg mice had more Tregs than WT mice throughout the infection, though the kinetics was similar (S2B Fig.). Besides, an increase in Tregs is associated with a decreased anti-viral immune response, which would not be in line with the increased protection we found in GITRL tg mice. Hence, it is most likely that GITR-mediated costimulation on Tregs does not have a major influence on T cell response to LCMV. Whether it does play a role in decreasing immunopathology in GITRL tg mice remains to be addressed.

The role of costimulation in immunity against chronic LCMV infection has also been examined for other TNFR family members and is highly diverse. Although similar, each of these molecules also has its own characteristic impact on cell type and effector function. Signaling through CD27 during acute and chronic LCMV infection enhances IFNγ and TNFα production by CD4^+^ T cells, but this actually contributes to pathology by inducing disruption of splenic architecture early during infection, which interferes with viral clearance by delaying the generation of virus-specific antibodies [14,57]. Costimulation through OX40 is required for optimal antiviral cellular and humoral immunity against LCMV Clone 13, but mice lacking OX40 had a much healthier appearance and lost significantly less weight than WT mice [58]. Interestingly, enhanced triggering through GITR boosted protective immunity to LCMV, and this was not accompanied by an expected increase in pathology, but rather by faster recovery of body weight and spleen cellularity and architecture. This would make GITR a very attractive target for boosting anti-viral immunity, as it would simultaneously prevent from tissue pathology.

Several preclinical studies in humans have shown that GITR-targeted therapies are effective in increasing the size and functionality of T cell response against different tumors. Most of these studies have used either the agonist antibody DTA-1 or GITRL-Fc molecules, although novel approaches with DNA and DC vaccines expressing GITRL have also been reported (reviewed in [18,19]). Interestingly, it has recently been shown that combined PD-1 blockade and GITR triggering can enhance anti-tumor immunity in murine cancer models, which can be further promoted with chemotherapeutic drugs [59], thus highlighting the potency of GITR stimulation also in combination therapy. The results presented in our current study open possibilities for targeting GITR in the treatment of chronic viral infections. In mouse models of chronic Friend virus (FV) infection, DTA-1 therapy during the acute phase of the infection produced faster Th1 immune responses and reduction in viral loads and pathology [60]. Although less marked, there was also improved CD8^+^ T cell function and reduction in viral loads after a combined transfer of transgenic CD8^+^ T cells and DTA-1 therapy in the chronic phase of FV infection [61]. In summary, the observations from this and previous papers suggest that GITR-targeted therapies could be used, in combination with other approaches, to restore function in exhausted CD8^+^ T cells during chronic viral infection without boosting immunopathology.

## Materials and Methods

### Animals and viruses

GITRL tg mice were maintained on a C57BL/6 background and bred in the animal department of the Academic Medical Center (Amsterdam, The Netherlands) under specific pathogen-free conditions. GITRL tg mice or their WT littermates were infected at 4–6 wk of age, age- and sex-matched within experiments, and were handled in accordance with institutional and national guidelines. LCMV clone 13 was grown in BHK-21 cells and tittered on Vero cells (both cell lines were kindly provided by Dr. E. John Wherry, University of Pennsylvania, USA), as previously described [62]. Mice were infected with 2×10^6^ PFU, i.v.

### Ethics statement

All mouse experiments were carried out in accordance with the Dutch Experiment on Animals Act and approved by the Animal Care and Use Committee of the University of Amsterdam (Permit numbers: DSK100401, DSK100044, DSK39 and DSK101745).

### Immunofluorescence microscopy

Spleens were formalin fixed, dehydrated in 30% sucrose solution and frozen in tissue tek embedding compound (Sakura Finetek, Torrance, CA). Sections of 5 μm were cut and stored at -20°C. Before staining sections were subjected to antigen retrieval with proteinase K (Roche, IN, USA) (3 min. 20 μg/ml in TE buffer pH 8.0 at RT) and blocked with 5% BSA/PBS. Sections were stained with rat-anti-MOMA-1 hybridoma supernatant (kind gift from Dr. Reina Mebius, VUMC, Amsterdam) 1:10 O/N at 4°C. As secondary antibody Alexa Fluor 647 conjugated donkey-anti-rat IgG was used (Jackson immunoresearch). Slides were blocked with 5% normal rat serum, 10 min RT and subsequently incubated with B220 Alexa Fluor 488 conjugated antibody (eBioscience) and Ter-119-biotinylated antibody (eBioscience), 1h RT. Finally sections were incubated with streptavidin Alexa Fluor 555 (Invitrogen). Hoechst was used as nuclear couterstain. Sections were mounted with Mowiol. Fluorescent images were obtained using a Zeiss Axio Examiner Z1 microscope.

### Quantitative real-time PCR

RNA was extracted using Trizol (Invitrogen) and complementary DNA was made with random hexamers and Superscript II reverse transcriptase (Roche). Quantitative real-time polymerase chain reaction (PCR) was performed in duplicate with Express SYBR GreenER reagents (Invitrogen) on the StepOnePlus RT-PCR system (Applied Biosystems), and data were normalized using HPRT as a reference gene. Primer sequences are available on request.

### In vivo CD4 depletion

To deplete CD4^+^ T cells before LCMV infection, mice were injected i.p. with 500 μg anti-CD4 antibody (clone GK1.5) on days-3 and 4 post-infection. In all cases, CD4 T cell depletion was confirmed via flow cytometry.

### ELISA

To quantify LCMV-specific antibodies, LCMV Clone 13 was used to coat 96-well Maxisorp ELISA plates (Nunc) overnight. Plates were blocked with 2% milk/PBS. Subsequently, serum isolated from the indicated mice was diluted 1/10 and then 3-fold serial dilutions were made. These dilutions were incubated on the LCMV-coated plates. Plates were subsequently incubated with a biotinylated donkey anti—mouse IgG antibody (Jackson immunoresearch), followed by a streptavidin-alkaline phosphatase conjugate (Jackson immunoresearch). p-Nitrophenyl phosphate (Sigma) was used as substrate. Optical density values were read using an ELISA plate reader (GENios Plus, Tecan) at 405nm and corrected at 550nm.

### Antibodies

The following mAbs from eBioscience were used: anti-CD44 (IM7), anti-CD4 (RM4–5), anti-CD8 (53–6.7), anti-CD62L (MEL-14), anti-B220 (RA3–6B2), anti-PD-1 (RMP1–30), anti-CD127 (A7R34), anti-GL-7 (GL-7), anti-CD40L (MR1), anti-Eomes (Dan11mag), anti-CD107α (1D4B) and anti-CD107β (ABL-93). From BioLegend: anti-KLRG1 (2F1), anti-CD3 (145–2C11), anti-SLAM (TC15–12F12.2); and from BD Biosciences: anti-Bcl6 (K112–91), anti-CXCR5 (RF8B2), anti-ICOS (7E.17G9), anti-FAS (Jo2), anti-CD138 (281–2), anti-CD25 (3C7), anti-IFN-γ (XMG1.2), anti-IL-2 (JES6–5H4), anti-TNF-α (MP6-XT22), anti-FoxP3 (MF23) and anti-T-bet (O4–46). Biotin conjugates were visualized by streptavidin-PE-Cy7 or streptavidin-eFluor 450 (eBioscience). Where possible, cells were stained in the presence of anti—CD16/CD32 block (2.4G2; kind gift from Louis Boon, Bioceros, Utrecht, The Netherlands) and dead cells were excluded by staining with LIVE/DEAD Fixable Near-IR Dead Cell Stain Kit (Invitrogen).

### Flow cytometry and intracellular cytokine staining

Lymphocytes were isolated from spleen, stained, and analyzed by flow cytometry. Virus-specific CD8^+^ or CD4^+^ T cells were examined with MHC class I or class II tetramers. MHC class I peptide tetramers for NP396 and GP33 peptides were made according to standard procedures [63], while the MHC class I tetramer for GP276 and the MHC class II tetramer for GP66 and its control with CLIP peptide were obtained from the NIH Tetramer Core Facility (Emory University, Atlanta, GA). For ICS, 2×10^6^ splenocytes were cultured in the presence or absence of peptide (2 μg/ml) and brefeldin A for 5 hr at 37C. Staining was carried out with the BD cytofix/cytoperm kit. Samples were collected by an LSR Fortessa or a Canto II flow cytometer (BD) and analyzed with FloJo software (Tree Star).

### Statistics

Mean values± SD are shown. Statistical analysis was performed using either 2-tailed Student t test, one-way or two-way ANOVA with GraphPad Prism 5 software.

## Supporting Information

S1 Fig Humoral responses in GITRL tg mice during chronic LCMV infections. WT (in white) and GITRL tg (in black) mice were intravenously infected with 2×10^6^ PFU of LCMV Cl13. (A) Absolute numbers of GL-7^+^FAS^+^B220^+^ GC B cells per spleen during the infection. (B) Representative FACS plot from (A) showing expression of FAS and GL-7 on B220^+^ cells in the spleen at day 15 p.i. Numbers represent % of GC B cells in the indicated gate. (C) Percentage of B220^lo^CD138^+^ plasma cells from total splenocytes at day 30 p.i. (D) ELISA for LCMV-specific IgG in serial dilution of serum at different days p.i. Results are shown as corrected OD (OD405-OD550 nm). Serum from non-infected mice was consistently <0.1 OD at 1:10 dilution for both genotypes.
Data are representative of 4–5 mice per group per time point. Error bars represent standard deviation. *p < 0.05, **p < 0.01, ***p < 0.001.
(PDF)

S2 Fig Treg and Tfr responses in GITRL tg mice during chronic LCMV infections. WT (in white) and GITRL tg (in black) mice were intravenously infected with 2×10^6^ PFU of LCMV Cl13. (A) Representative FACS plots showing CD4 vs FoxP3 expression of CD4^+^CD3^+^ cells from spleens of WT (top) or GITRL tg (bottom) mice. (B) % FoxP3^+^ cells of CD4^+^CD3^+^ cells throughout infection with LCMV. (C) Representative FACS plots showing CXCR5 vs FoxP3 expression of CD4^+^CD3^+^ cells from spleens of WT (top) or GITRL tg (bottom) mice. (D) *Top*: Representative histograms showing CD25 expression of FoxP3^+^ CD4^+^CD3^+^ cells from spleens of WT (black line) or GITRL tg (grey filled curve) mice. *Bottom*: geometric MFI of CD25 in FoxP3^+^ CD4^+^CD3^+^ cells. Data are representative of 4–5 mice per group per time point. Error bars represent standard deviation. **p < 0.01, ***p < 0.001.
(PDF)

S3 Fig Treg and Tfr responses in GITRL tg mice during chronic LCMV infections. WT (in white) and GITRL tg (in black) mice were intravenously infected with 2×10^6^ PFU of LCMV Cl13. (A) Representative FACS plots showing either T-bet (left) or Eomes (right) vs I-A^b^GP_66_ tetramer staining of CD4^+^CD3^+^ cells from spleens of WT (top) or GITRL tg (bottom) mice at day 8 p.i. (B) *Left*: Representative FACS plots showing Eomes vs T-bet expression in total CD4^+^CD3^+^ cells from spleens of WT (top) or GITRL tg (bottom) mice at day 8 p.i. *Right*: % Eomes^-^ T-bet^+^ (top) or % Eomes^+^ T-bet^-^ (bottom) of total CD4^+^CD3^+^ cells. (C) Representative FACS plots showing Eomes vs T-bet expression in I-A^b^GP_66_ tetramer^+^ CD4^+^CD3^+^ cells from spleens of WT (top) or GITRL tg (bottom) mice at day 8 p.i. Data are representative of 4–5 mice per group per time point. Error bars represent standard deviation. **p < 0.01, ***p < 0.001, ****p < 0.0001.
(PDF)

S4 Fig NK cells in GITRL tg mice during steady state and LCMV infections. WT (in white) and GITRL tg (in black) mice were either analyzed in the steady state (A-D) or intravenously infected with 2×10^6^ PFU of LCMV Cl13 and analyzed at day 8 p.i. (E-H). (A&E) *Left*: Representative FACS plots showing CD3 vs NK1.1 stainings in total nucleated cells form spleens of WT (top) or GITRL tg (bottom) mice in the steady state. *Center*: % CD3^-^ NK1.1^+^ cells of total nucleated spleen cells. *Right*: Absolute numbers of CD3^-^ NK1.1^+^ cells per spleen. (B&F) *Left*: Representative histogram showing DX5 expression of CD3^-^ NK1.1^+^ cells from spleens of WT (top) or GITRL tg (bottom) mice. *Right*: % DX5^+^ of CD3^-^ NK1.1^+^ cells. (C&G) *Left*: Representative FACS plots showing KLRG1 vs Granzyme B expression of CD3^-^ NK1.1^+^ cells. In panel C, CD3^-^ NK1.1^-^ cells are overlayed in blue for comparison. Right: geometric MFI of Granzyme B (top) and % KLRG1+ cells (bottom) among CD3^-^ NK1.1^+^ cells. (D&H) *Left*: Representative FACS plots showing CD11b vs CD27 stainings in total nucleated cells form spleens of WT (top) or GITRL tg (bottom) mice in the steady state. *Right*: % of the different populations within CD3^-^ NK1.1^+^ cells. Data are representative of 4–5 mice per group per condition. Error bars represent standard deviation. *p < 0.05, **p < 0.01, ***p < 0.001.
(PDF)

S5 Fig GITR expression on CD4 and CD8 T cells during steady state and LCMV infections. WT (in white) and GITRL tg (in black) mice were either analyzed in the steady state (A) or intravenously infected with 2×10^6^ PFU of LCMV Cl13 and analyzed at day 8 p.i. (B). Results show the geometric MFI of GITR within different populations of cells. Data are representative of 4–5 mice per group per condition. Error bars represent standard deviation. **p < 0.01.
(PDF)
