# Supplementary figures and images for: Enhanced CD8 T Cell Responses through GITR-Mediated Costimulation Resolve Chronic Viral Infection
Source: PLoS Pathog. 2015 Mar 4;11(3):e1004675. doi: 10.1371/journal.ppat.1004675 (PMC4349659; doi:10.1371/journal.ppat.1004675)

**Supplementary Figure 1:** Humoral responses in GITRL tg mice during chronic LCMV infection.

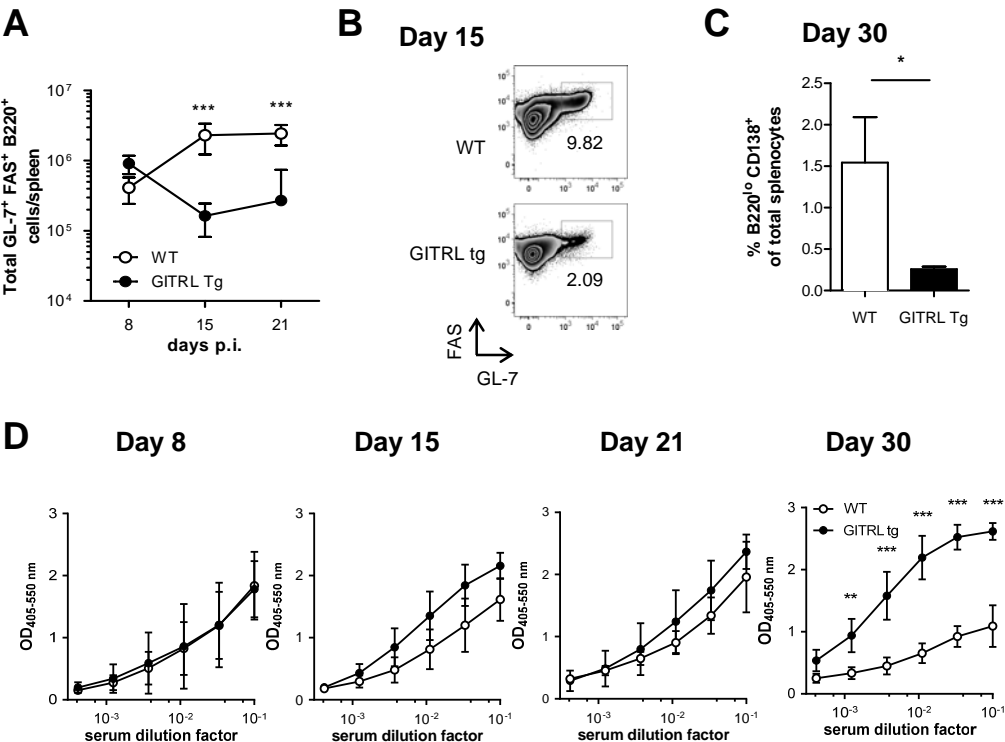

Supplement: S1 Fig — WT (in white) and GITRL tg (in black) mice were intravenously infected with 2×10^6^ PFU of LCMV Cl13. (A) Absolute numbers of GL-7+FAS+B220+ GC B cells per spleen during the infection. (B) Representative FACS plot from (A) showing expression of FAS and GL-7 on B220+ cells in the spleen at day 15 p.i. Numbers represent % of GC B cells in the indicated gate. (C) Percentage of B220loCD138+ plasma cells from total splenocytes at day 30 p.i. (D) ELISA for LCMV-specific IgG in serial dilution of serum at different days p.i. Results are shown as corrected OD (OD405-OD550 nm). Serum from non-infected mice was consistently <0.1 OD at 1:10 dilution for both genotypes. Data are representative of 4–5 mice per group per time point. Error bars represent standard deviation. *p < 0.05, **p < 0.01, ***p < 0.001. (PDF) [file ppat.1004675.s001.pdf]

**Supplementary Figure 2:** Treg and Tfr responses in GITRL tg mice during chronic LCMV infection.

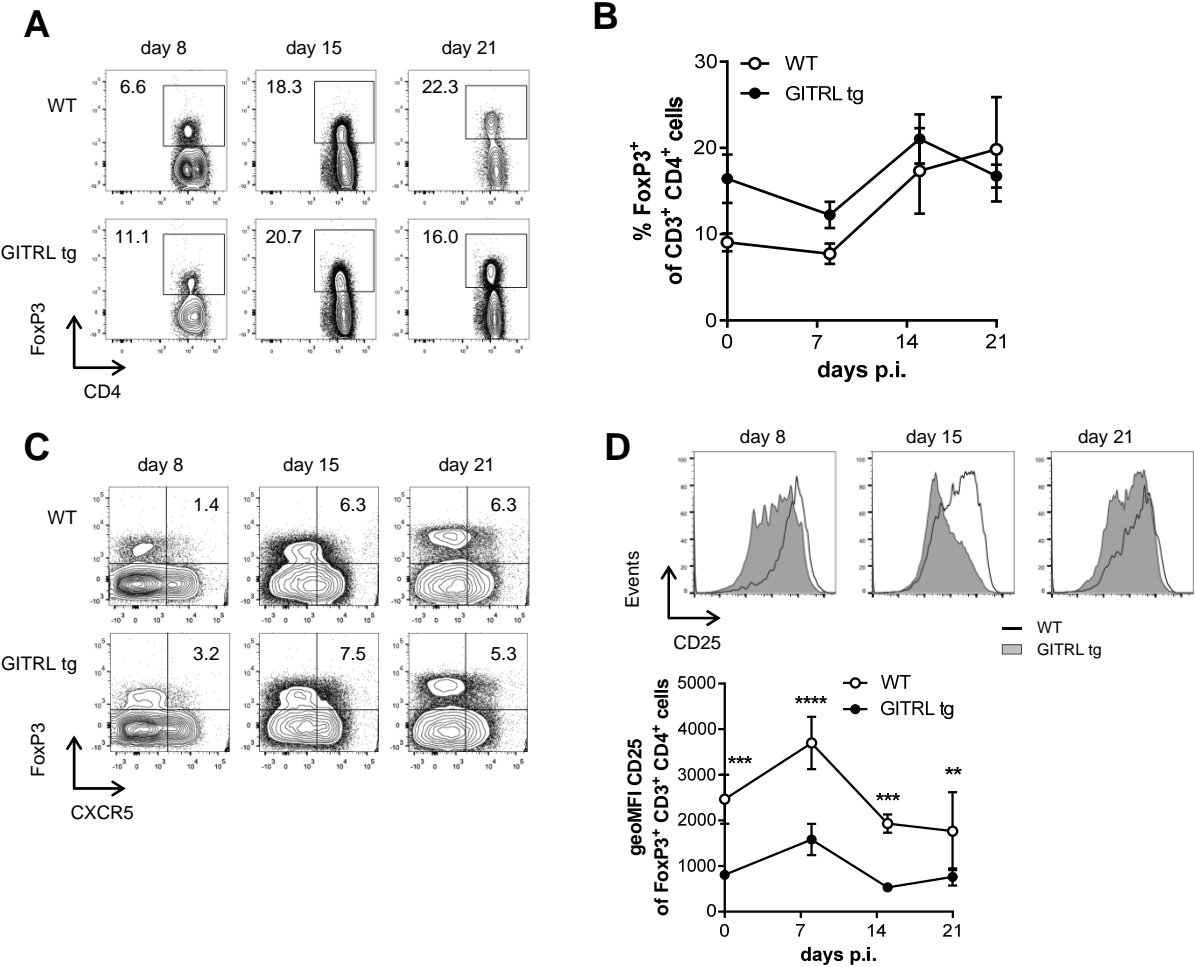

Supplement: S2 Fig — WT (in white) and GITRL tg (in black) mice were intravenously infected with 2×10^6^ PFU of LCMV Cl13. (A) Representative FACS plots showing CD4 vs FoxP3 expression of CD4+CD3+ cells from spleens of WT (top) or GITRL tg (bottom) mice. (B) % FoxP3+ cells of CD4+CD3+ cells throughout infection with LCMV. (C) Representative FACS plots showing CXCR5 vs FoxP3 expression of CD4+CD3+ cells from spleens of WT (top) or GITRL tg (bottom) mice. (D) Top: Representative histograms showing CD25 expression of FoxP3+ CD4+CD3+ cells from spleens of WT (black line) or GITRL tg (grey filled curve) mice. Bottom: geometric MFI of CD25 in FoxP3+ CD4+CD3+ cells. Data are representative of 4–5 mice per group per time point. Error bars represent standard deviation. **p < 0.01, ***p < 0.001. (PDF) [file ppat.1004675.s002.pdf]

**Supplementary Figure 3:** Expression of TFs in CD4<sup>+</sup> cells during LCMV infection.

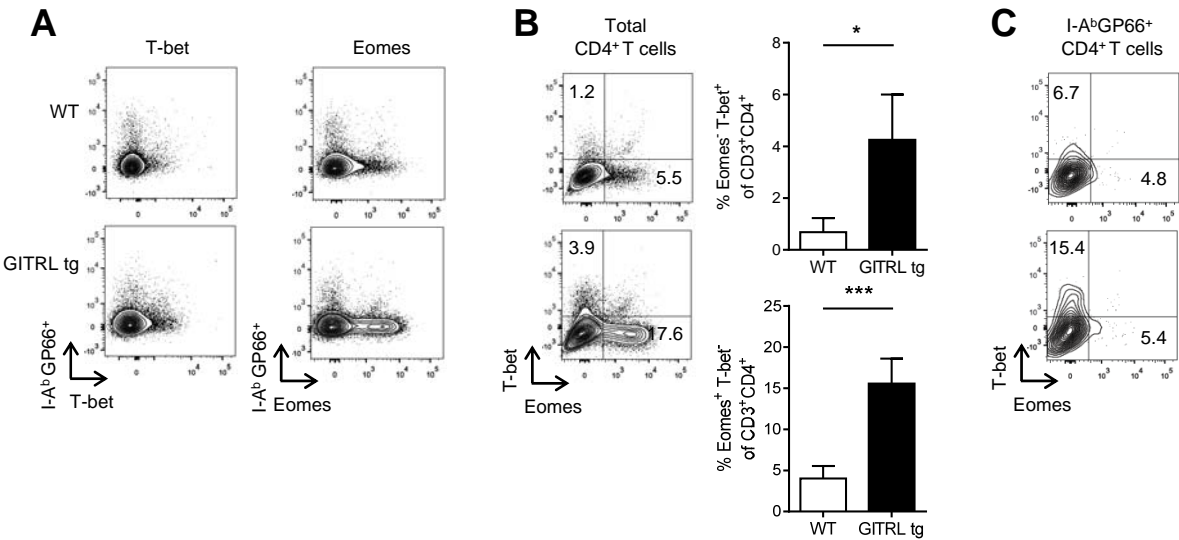

Supplement: S3 Fig — WT (in white) and GITRL tg (in black) mice were intravenously infected with 2×10^6^ PFU of LCMV Cl13. (A) Representative FACS plots showing either T-bet (left) or Eomes (right) vs I-AbGP66 tetramer staining of CD4+CD3+ cells from spleens of WT (top) or GITRL tg (bottom) mice at day 8 p.i. (B) Left: Representative FACS plots showing Eomes vs T-bet expression in total CD4+CD3+ cells from spleens of WT (top) or GITRL tg (bottom) mice at day 8 p.i. Right: % Eomes- T-bet+ (top) or % Eomes+ T-bet- (bottom) of total CD4+CD3+ cells. (C) Representative FACS plots showing Eomes vs T-bet expression in I-AbGP66 tetramer+ CD4+CD3+ cells from spleens of WT (top) or GITRL tg (bottom) mice at day 8 p.i. Data are representative of 4–5 mice per group per time point. Error bars represent standard deviation. **p < 0.01, ***p < 0.001, ****p < 0.0001. (PDF) [file ppat.1004675.s003.pdf]

**Supplemental Figure 4: NK cells in GITRL tg mice during steady state and LCMV infection.**

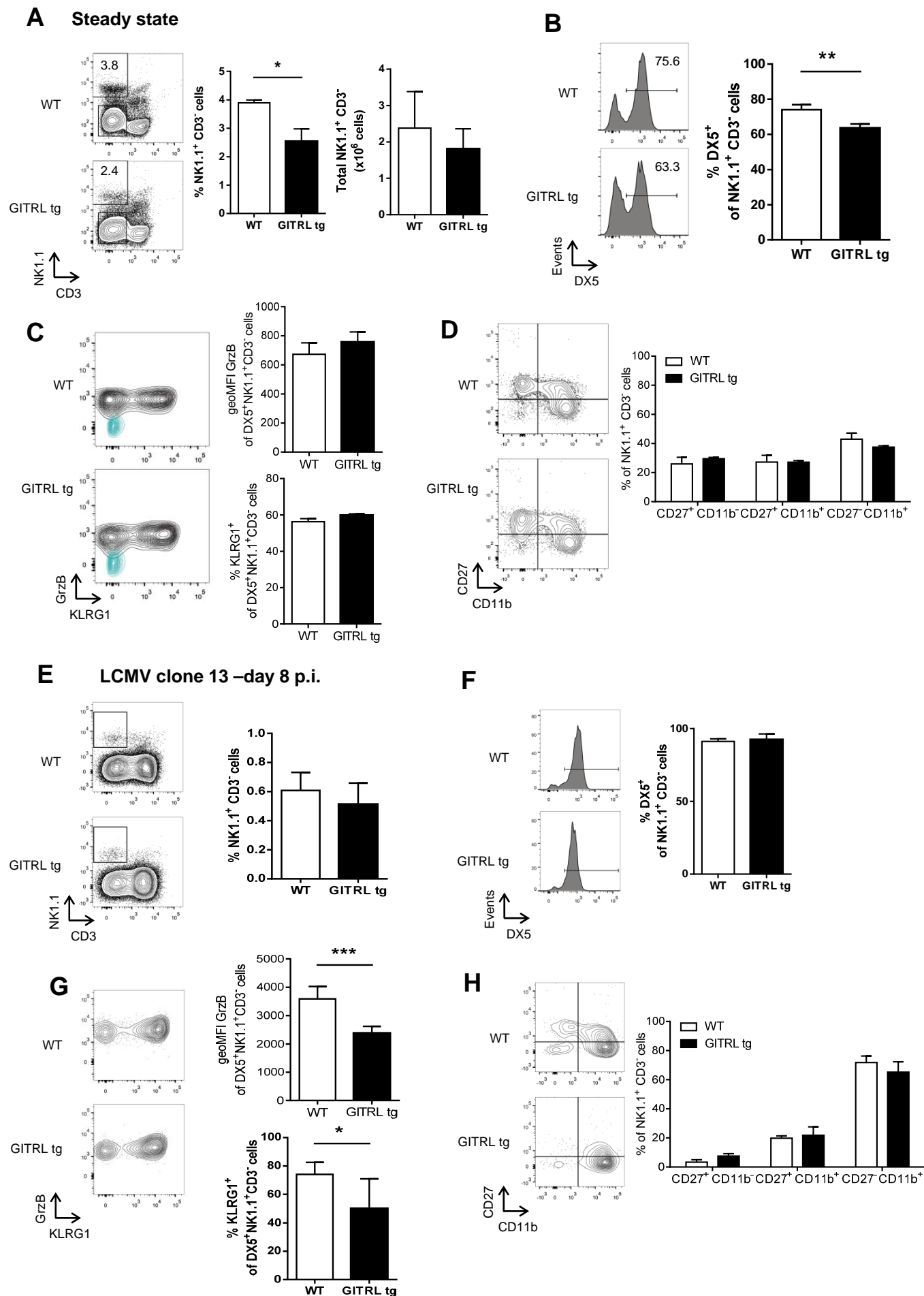

Supplement: S4 Fig — WT (in white) and GITRL tg (in black) mice were either analyzed in the steady state (A-D) or intravenously infected with 2×10^6^ PFU of LCMV Cl13 and analyzed at day 8 p.i. (E-H). (A&E) Left: Representative FACS plots showing CD3 vs NK1.1 stainings in total nucleated cells form spleens of WT (top) or GITRL tg (bottom) mice in the steady state. Center: % CD3- NK1.1+ cells of total nucleated spleen cells. Right: Absolute numbers of CD3- NK1.1+ cells per spleen. (B&F) Left: Representative histogram showing DX5 expression of CD3- NK1.1+ cells from spleens of WT (top) or GITRL tg (bottom) mice. Right: % DX5+ of CD3- NK1.1+ cells. (C&G) Left: Representative FACS plots showing KLRG1 vs Granzyme B expression of CD3- NK1.1+ cells. In panel C, CD3- NK1.1- cells are overlayed in blue for comparison. Right: geometric MFI of Granzyme B (top) and % KLRG1+ cells (bottom) among CD3- NK1.1+ cells. (D&H) Left: Representative FACS plots showing CD11b vs CD27 stainings in total nucleated cells form spleens of WT (top) or GITRL tg (bottom) mice in the steady state. Right: % of the different populations within CD3- NK1.1+ cells. Data are representative of 4–5 mice per group per condition. Error bars represent standard deviation. *p < 0.05, **p < 0.01, ***p < 0.001. (PDF) [file ppat.1004675.s004.pdf]

**Supplemental Figure 5:** Expression of GITR during steady state and LCMV infection.

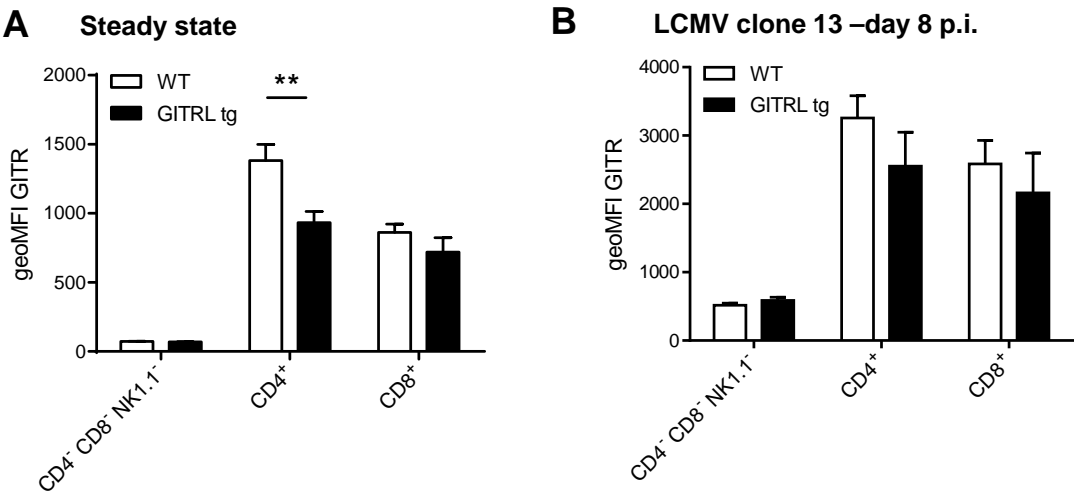

Supplement: S5 Fig — WT (in white) and GITRL tg (in black) mice were either analyzed in the steady state (A) or intravenously infected with 2×10^6^ PFU of LCMV Cl13 and analyzed at day 8 p.i. (B). Results show the geometric MFI of GITR within different populations of cells. Data are representative of 4–5 mice per group per condition. Error bars represent standard deviation. **p < 0.01. (PDF) [file ppat.1004675.s005.pdf]
